# UN Sustainable Development Goals: establishment of an electronic ‘collection’ of papers published in Radiation and Environmental Biophysics

**DOI:** 10.1007/s00411-023-01028-1

**Published:** 2023-05-10

**Authors:** Werner Rühm, Anna A. Friedl, Andrzej Wojcik

**Affiliations:** 1 Federal Office for Radiation Protection, München (Neuherberg), Germany; 2 Department of Radiation Oncology, KUM University Hospital, LMU University of Munich, Munich, Germany; 3 Centre for Radiation Protection Research, Stockholm University, Svante Arrheniusväg 20C, 106 91 Stockholm, Sweden; 4 Institute of Biology, Jan Kochanoski University, 25-406 Kielce, Poland

At the 70th session in September 2015 in New York, the member states of the United Nations adopted a resolution on “Transforming our World: the 2030 Agenda for Sustainable Development” (UN General Assembly 2015). The document is considered as a “shared blueprint for peace and prosperity for people and the planet, now and into the future” (https://sdgs.un.org/goals). The resolution includes 17 so-called “Sustainable Development Goals” (SDGs) complemented by 169 Targets, and basically represents a call for action to fight poverty together with improving health and education, reducing inequality and supporting economic growth. In doing so, the environment including oceans and forests should be preserved and challenges due to climate change be addressed.

Those 17 SDGs are:

- SDG #1: end poverty in all its forms everywhere.
- SDG #2: end hunger, achieve food security and improved nutrition and promote sustainable agriculture.
- SDG #3: ensure healthy lives and promote well-being for all at all ages.
- SDG #4: ensure inclusive and equitable quality education and promote lifelong learning opportunities for all.
- SDG #5: achieve gender equality and empower all women and girls.
- SDG #6: ensure availability and sustainable management of water and sanitation for all.
- SDG #7: ensure access to affordable, reliable, sustainable and modern energy for all.
- SDG #8: promote sustained, inclusive and sustainable economic growth, full and productive employment and decent work for all.
- SDG #9: build resilient infrastructure, promote inclusive and sustainable industrialization and foster innovation.
- SDG #10: reduce inequality within and among countries.
- SDG #11: make cities and human settlements inclusive, safe, resilient and sustainable.
- SDG #12: ensure sustainable consumption and production patterns.
- SDG #13: take urgent action to combat climate change and its impacts.
- SDG #14: conserve and sustainably use the oceans, seas and marine resources for sustainable development.
- SDG #15: protect, restore and promote sustainable use of terrestrial ecosystems, sustainably manage forests, combat desertification, and halt and reverse land degradation and halt biodiversity loss.
- SDG #16: promote peaceful and inclusive societies for sustainable development, provide access to justice for all and build effective, accountable and inclusive institutions at all levels.
- SDG #17: strengthen the means of implementation and revitalize the Global Partnership for Sustainable Development.

Independent Group of Scientists appointed by the Secretary-General (2019) emphasized the importance of science in the implementation of the 2030 Agenda across the social, economic and environmental dimensions of sustainable development.

Given the overarching importance of sustainability for the conservation of our planet and for the survival of mankind, Springer Nature considers means to support the research community in their efforts to contribute reaching these SDGs. Specifically, Springer Nature has established an SDG Programme that aims to connect scientists working on solutions to achieve these goals with people working in policy, industry and business who need solutions for implementing SDGs. The Programme includes publishing relevant activities more visible to key communities through a variety of channels (www.springernature.com/gp/researchers/sdg-programme). To quote Sir Philip Campbell, Editor-in-Chief of Springer Nature: “The themes of the UN Sustainable Development Goals are inspirational to us at Springer Nature. Across our publishing and services and across the disciplines, we are focusing on helping researchers make the world a better place for future generations”.

Now, what does this all mean for Radiation and Environmental Biophysics? Is there anything we can do to support our authors and readers, should they have the desire and means to provide support in reaching the UN SDGs? Together with Springer Nature, we have decided to establish an Electronic ‘Collection’ (Special Issue) that will include publications relevant for the UN SDGs. Upon acceptance, the papers accepted will be published in the Collection. Submission is open without a deadline. Authors are invited to submit papers related to the use of ionizing radiation or to radiation protection, if they feel that they address one or more of the 17 UN SDGs. Of course, every submitted paper will undergo our regular peer-review process to keep the high scientific quality of publications in REBS. Should a paper initially submitted for the Collection and be considered outside the remit of the Collection, then it may still be considered for a ‘regular’ issue.

The first paper of—hopefully—many more to be published in our Collection on the UN SDGs deals with a recent initiative of the International Commission on Radiological Protection (www.icrp.org). At the last ICRP Symposium in October 2022 in Vancouver, Canada, a Call for Action was announced to strengthen expertise in radiological protection worldwide. The call addressed national governments and funding agencies to strengthen resources for radiological protection research, national research laboratories and other institutions to launch and sustain long-term research programs, and universities to develop undergraduate and graduate programs and make students aware of job opportunities in radiation-related fields. Furthermore, ICRP proposed/urged to use plain language when interacting with the public and decision-makers about radiological protection, and to foster general awareness of radiation and radiological protection through education and training of information multipliers. In the Call, explicit reference is made to the SDG #3 “Good Health and Well-being”, #4 “Quality Education”, #10 “Reduced Inequalities”, #14 “Life Below Water” and #15 “Life on Land” (Rühm et al. 2023).

With this initiative, we strongly support Springer Nature in promoting the UN SDGs, and we gladly accept the offer of Springer Nature to Radiation and Environmental Biophysics, to take a lead role within the Biophysics journal portfolio of our publisher. Ionizing radiation is a double-edged sword: it is an indispensable tool in medicine and industry but its use, if not justified and safe, can impair health. It is up to our authors to demonstrate that the international scientific community working in radiation research and radiation protection can contribute through innovation to a sustainable development of our planet, for the benefit of current and future generations.
